# The herpes simplex virus UL20 protein functions in glycoprotein K (gK) intracellular transport and virus-induced cell fusion are independent of UL20 functions in cytoplasmic virion envelopment

**DOI:** 10.1186/1743-422X-4-120

**Published:** 2007-11-08

**Authors:** Jeffrey M Melancon, Preston A Fulmer, Konstantin G Kousoulas

**Affiliations:** 1 Division of Biotechnology and Molecular Medicine, School of Veterinary Medicine, Louisiana State University, Baton Rouge, USA

## Abstract

The HSV-1 UL20 protein (UL20p) and glycoprotein K (gK) are both important determinants of cytoplasmic virion morphogenesis and virus-induced cell fusion. In this manuscript, we examined the effect of UL20 mutations on the coordinate transport and Trans Golgi Network (TGN) localization of UL20p and gK, virus-induced cell fusion and infectious virus production. Deletion of 18 amino acids from the UL20p carboxyl terminus (UL20 mutant 204t) inhibited intracellular transport and cell-surface expression of both gK and UL20, resulting in accumulation of UL20p and gK in the endoplasmic reticulum (ER) in agreement with the inability of 204t to complement UL20-null virus replication and virus-induced cell fusion. In contrast, less severe carboxyl terminal deletions of either 11 or six amino acids (UL20 mutants 211t and 216t, respectively) allowed efficient UL20p and gK intracellular transport, cell-surface expression and TGN colocalization. However, while both 211t and 216t failed to complement for infectious virus production, 216t complemented for virus-induced cell fusion, but 211t did not. These results indicated that the carboxyl terminal six amino acids of UL20p were crucial for infectious virus production, but not involved in intracellular localization of UL20p/gK and concomitant virus-induced cell fusion. In the amino terminus of UL20, UL20p mutants were produced changing one or both of the Y38 and Y49 residues found within putative phosphorylation sites. UL20p tyrosine-modified mutants with both tyrosine residues changed enabled efficient intracellular transport and TGN localization of UL20p and gK, but failed to complement for either infectious virus production, or virus-induced cell fusion. These results show that UL20p functions in cytoplasmic envelopment are separable from UL20 functions in UL20p intracellular transport, cell surface expression and virus-induced cell fusion.

## Introduction

Herpes simplex viruses (HSV) specify at least eleven virus-specified glycoproteins, as well as several non-glycosylated membrane associated proteins, most of which play important roles in multiple membrane fusion events during virus entry and intracellular virion morphogenesis and egress [1-8]. Spread of infectious virus occurs either by release of virions to extracellular spaces or through virus-induced cell-to-cell fusion. In vivo, the latter mechanism allows for virus spread without exposing virions to extracellular spaces containing neutralizing antibodies. Mutations that cause extensive virus-induced cell fusion predominantly arise in four genes of the HSV genome: the UL20 gene [9,10], the UL24 gene [11,12], the UL27 gene encoding glycoprotein B (gB) [13,14], and the UL53 gene coding for glycoprotein K (gK) [15-19]. Of these four membrane associated proteins, only UL20 and gK are absolutely essential for the intracellular envelopment and transport of virions to extracellular spaces in all cell types [9,20-23].

The most prevalent model for morphogenesis and egress of infectious herpes virions includes sequential de-envelopment and re-envelopment steps in transit to extracellular spaces: a) primary envelopment by budding of capsids assembled in the nuclei through the inner nuclear leaflet leading to the production of enveloped virions within perinuclear spaces; b) de-envelopment by fusion of viral envelopes with the outer nuclear leaflet leading to the accumulation of unenveloped capsids in the cytoplasm; c) assembly of sets of tegument proteins on the cytoplasmic capsids, as well as potentially on vesicle sites to be used for cytoplasmic envelopment; d) re-envelopment of cytoplasmic tegumented capsids into TGN-derived vesicles. This final event in cytoplasmic virion envelopment is thought to be largely mediated by interactions between tegument proteins and cytoplasmic portions of viral glycoproteins embedded within the TGN-derived membranes. Cytoplasmically enveloped viruses are thought to be transported to extracellular spaces within Golgi or TGN-derived vesicles (reviewed in: [7,24,25]).

The UL20 gene encodes a 222 amino acid non-glycosylated transmembrane protein that is conserved by all alphaherpesviruses. The UL20p is a structural component of extracellular enveloped virions and it is expressed in infected cells assuming a predominantly perinuclear and cytoplasmic distribution [26]. An initial report indicated that partial deletion of the UL20 gene resulted in perinuclear accumulation of capsids indicating that the UL20 gene functioned, most likely, in the de-envelopment of enveloped virions found within perinuclear spaces [9]. However, we showed previously that a precise deletion of the UL20 gene revealed that the UL20 gene strictly functioned in cytoplasmic envelopment of capsids [27]. Importantly, syncytial mutations in either gB or gK failed to cause fusion in the absence of the UL20 gene, indicating that the UL20 protein was essential for virus-induced cell fusion [27]. Furthermore, we showed that UL20 is required for cell-surface expression of gK and TGN localization, suggesting a functional interdependence between gK and UL20 for virus egress and cell-to-cell fusion [28,29]. Recently, we delineated via site-directed mutagenesis the functional domains of UL20p involved in infectious virus production and virus-induced cell fusion. Importantly, we showed that both amino and carboxyl terminal portions of UL20p, which are predicted to lie within the cytoplasmic side of cellular membranes, function both in cytoplasmic virion envelopment and virus-induced cell fusion [30].

In this manuscript, we show that the amino and carboxyl termini of UL20p contain distinct domains that function in infectious virion production and intracellular gK/UL20 transport.

## Results

### Mutagenesis of HSV-1 UL20

**Figure 1:**
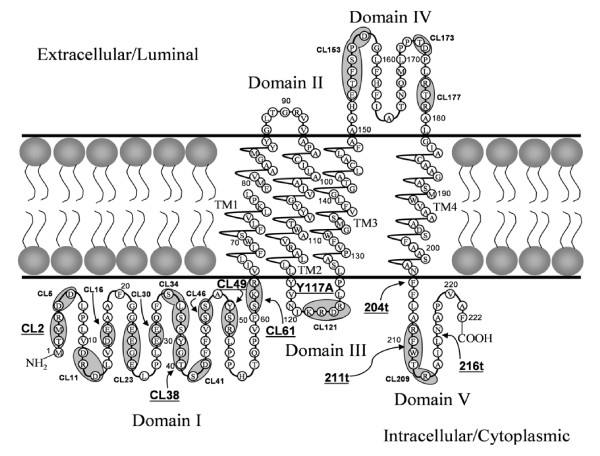
**Predicted membrane topology of UL20p of UL20 mutations described previously [30, 31] (small fonts), and new and other UL20 mutations discussed in this manuscript (larger fonts, underlined)**. UL20p domains where cluster-to-alanine mutations are located are indicated by a shaded oval. Naming of cluster mutations is based on the first amino acid mutated in each cluster. Single amino acid replacements are indicated with the amino acid position bracketed on the left by the targeted amino acid and on the right by the changed amino acid i.e. Y38A. Carboxyl terminal truncations are indicated by the let (t) following the terminal amino acid of the truncated UL20p. Transmembrane region (TM), Cluster mutant (CL).

Previously, we reported on the construction and characterization of a panel of 31 mutations within the UL20 gene [30]. These mutations included: 1) cluster-to-alanine mutants in which a cluster of proximal amino acids were changed to alanine residues; 2) single amino acid replacement mutants within alanine cluster regions; 3) carboxyl terminal truncations of UL20p. Two additional double mutants where constructed for the present study. UL20 mutant CL38 – CL49 combined the two cluster mutations targeting the two putative phosphorylation sites in the amino terminus of UL20p. Similarly, the Y38A – Y49A double mutant combined the two specific tyrosine modifications without altering adjacent amino acids. In addition, UL20 mutants CL2, CL61, Y38A, and Y117A, which were not reported previously, were included in these investigations. All UL20 mutants were tested for their ability to complement UL20-null infectious virus production, as well as either gB or gK-mediated virus-induced cell fusion having the gBsyn3, or gKsyn1 mutation, respectively. The mutated amino acids for each type of mutation included in this study are shown in Table 1. The constructed UL20 carboxyl terminal truncations are identified with the number of the last remaining amino acid (i.e. 204t retains UL20p amino acids 1–204). The location of each mutation with respect to the UL20p topology [30] is shown in Figure 1.

**Table 1:** 

**Domain**	**Mutation Name**	**WT aa Sequence**	**Mut. aa Sequence**
I	CL38	YGT	AGA
I	CL49	YSR	AAA
I	Y38A	YGT	AGT
I	Y49A	YSR	ASR
I	CL38-CL49	YGT-YSR	AGA-AAA
I	Y38A-Y49A	YGT-YSR	AGT-ASR
I	CL61	SKR	SKA
IV	CL153	ETFSPD	AAFAPA
V, C-Truncation	204t	SANFF	SANG
V, C-Truncation	211t	RFWTR	RFWG*
V, C-Truncation	216t	AILNA	AILG*

*Indicates stop codon

### Complementation assay for infectious virus production

**Figure 2:**
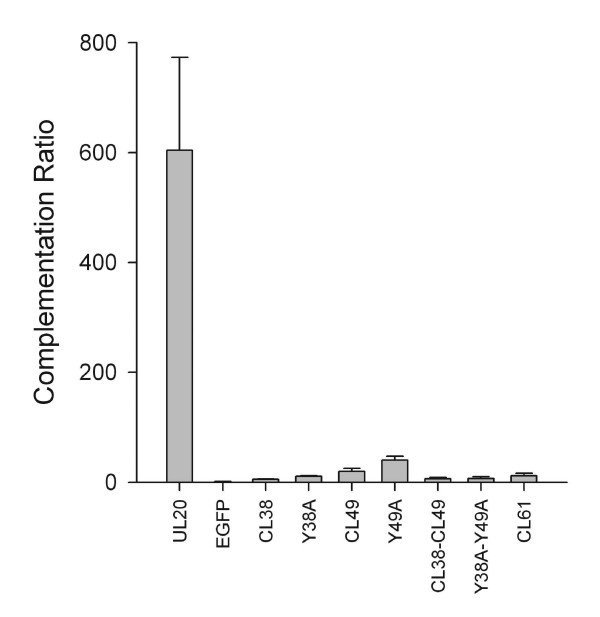
**Complementation ratios produced by mutant UL20p genes**. Vero cells were transfected with plasmids encoding wild-type or mutant UL20 genes under the UL20 promoter and then infected with the HSV-1(KOS) UL20-null (Δ20) virus. Viral stocks were prepared at 24 hours post infection and tittered on Vero cells (see Materials & Methods). The error bars shown represent the maximum and minimum complementation ratios obtained from three independent experiments, and the bar height represents the average complementation ratio.

It was previously shown that deletion of the HSV-1 UL20 and the PRV UL20 genes resulted in up to two logs reduction in infectious virus production relative to their parental wild type strains. The targeted set of single or double UL20 mutants and UL20p truncations were tested for their ability to complement the HSV-1(KOS) UL20-null virus. Complementation experiments involved transfection of Vero cells with plasmids encoding wild-type or mutant UL20 genes, followed by infection with the UL20-null virus as reported previously [27,30] and described in Materials and Methods. A complementation ratio was calculated for each mutant UL20 plasmid as a percent ratio to complementation levels provided by the wild-type UL20 gene. The UL20 wild-type gene effectively complemented UL20-null virus infectious virus production, while the UL20 mutants targeted in this study failed to complement the UL20-null virus (Fig. 2). Furthermore, the CL2 and Y117A mutations complemented the UL20-null virus to wild-type levels (not shown).

### Complementation for virus-induced cell-to-cell fusion

**Figure 3:**
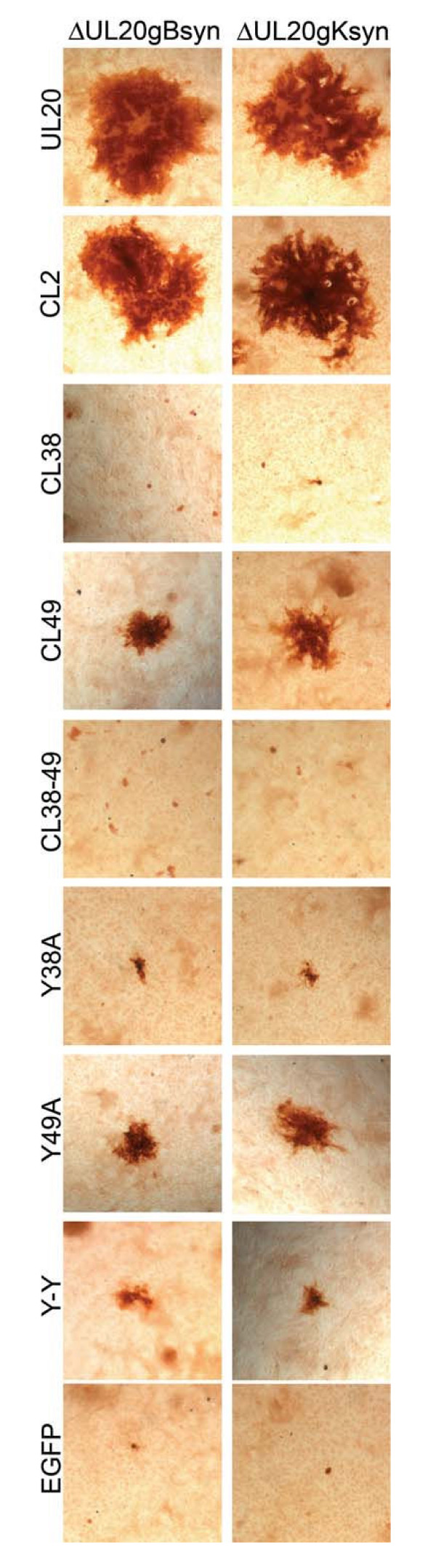
**Plaque phenotypes of representative viral plaques obtained after rescue of the Δ20 gK, Δ20 gK syn1, or Δ20 gKsyn3 viruses**. Vero cell monolayers were transfected with plasmids expressing the mutant UL20 genes, and 24 hours later, they were infected with the respective Δ20 gK-null viruses carrying either the syn1 (gK) or gB(syn3) mutation. Viral plaques were visualized by immunohistochemistry at 24 hpi.

We previously showed that syncytial mutations in either gB or gK failed to cause virus-induced cell fusion in the absence of the UL20 gene [27]. Furthermore, a panel of 31 different UL20 mutants revealed that UL20 domains that functioned in infectious virus production segregated from those that functioned in virus-induced cell fusion [30]. The panel of UL20 mutants shown in Table 1 was tested for the ability to complement UL20-null viruses containing syncytial mutations in either gB (syn3) or gK (syn1) for virus-induced cell fusion as described previously [30]. Briefly, confluent Vero monolayers were transfected with plasmids encoding either wild type or mutant UL20p, and subsequently infected with either Δ20 gKsyn1 or Δ20 gBsyn3 viruses. Viral plaques appearing as larger plaques in a background of uniformelly small UL20-null viral plaques were stained with anti-HSV-1 polyclonal antibody as described in Materials and Methods (Fig. 3). In this complementation assay, 20–40% of all viral plaques appeared considerably larger than the uniformly small UL20-null plaques (not shown). The CL2 UL20 mutant (Fig. 3) and Y117A (not shown) complemented effectively both gB and gK-mediated virus-induced cell fusion, as evidenced by the appearance of viral plaques similar in size to those produced by the wild-type UL20 gene. As previously described [30], and as shown here, the CL49 and Y49A mutations partially complemented virus spread and virus-induced cell fusion caused by syncytial mutations in either gB or gK, as evidenced by the production of visibly larger than the UL20-null viral plaques (Fig. 3). The CL38, Y38A, and the double mutants CL38-CL39 and Y38A-Y39A failed to complement for either infectious virus production or virus spread, as evidenced by the appearance of very small viral plaques (Fig. 3). These results confirmed the complementation for infectious virus production results shown in figure 2.

### Intracellular transport and TGN localization of UL20p mutants and gK

**Figure 4:**
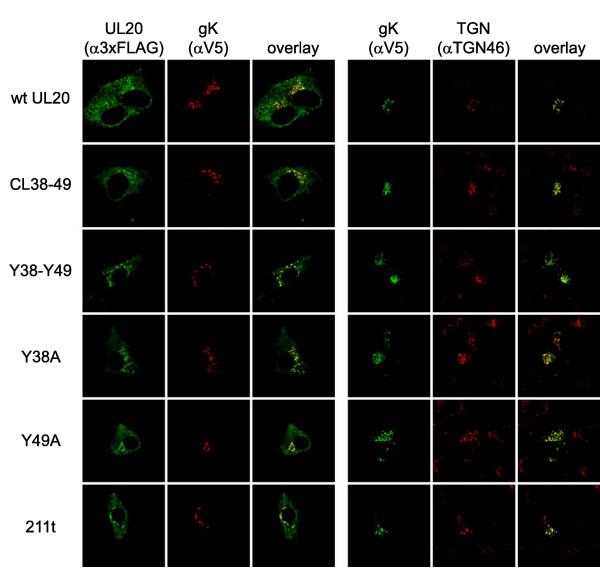
**The effect of UL20p amino terminal mutations on UL20p and gK colocalization in TGN cellular compartments**. Vero cells were co-transfected with gK tagged with the V5 epitope (D1V5), as well as with plasmids encoding wild-type or different mutant UL20ps tagged with the 3 × FLAG epitope (UL20D1FLAG). Thirty-six hours post-transfection, cells were washed thoroughly, fixed, and processed for confocal microscopy. After permeabilization, rabbit anti-FLAG (α FLAG) mAb was used to detect UL20p, mouse anti-V5 (α V5) epitope was used to detect gK, and sheep aTGN46 mAb was used to identify the TGN. First three rows of the confocal pictures show co-localization of UL20p with gK, while rows 4–6 show colocalization of gK with TGN46.

**Figure 5:**
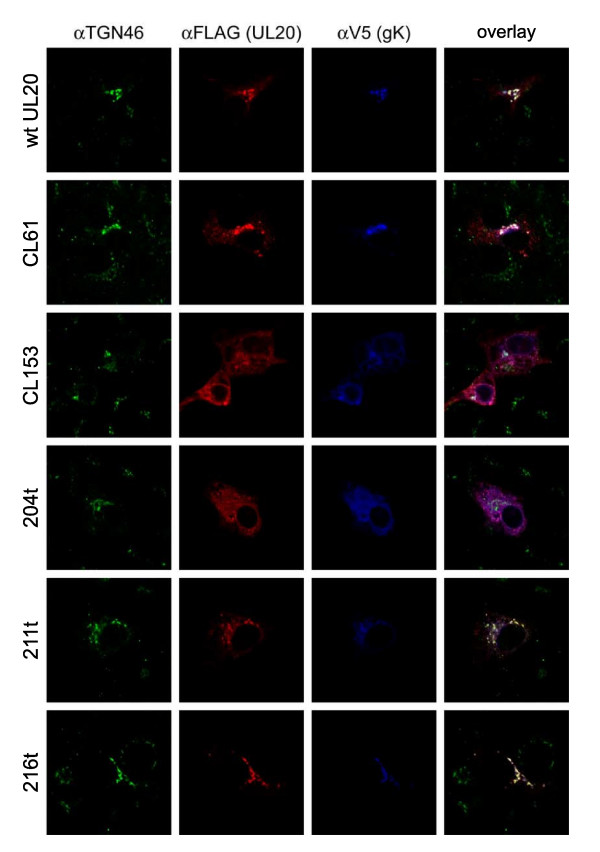
**The effect of UL20p carboxyl terminal truncations on UL20p and gK colocalization in TGN cellular compartments**. As with figure 4, Vero cells were co-transfected with gKD1V5, as well as with plasmids encoding wild-type or mutant UL20DIFLAG proteins. Thirty-six hours post-transfection, cells were washed thoroughly, fixed, and processed for confocal microscopy. After permeabilization, antibodies a3xFLAG, aV5 and aTGN46 were used to identify, UL20p, gK and TGN46, respectively.

Transport and localization of UL20p and gK was further assessed by transient coexpression of gK and UL20p and simultaneous detection of the TGN compartment. We showed previously that in the absence of UL20p, gK was localized exclusively to reticular-like compartments and was absent from the Golgi and TGN. A similar pattern was detected for UL20p in the absence of gK [31]. In contrast, coexpression of gK and UL20p significantly altered the distribution pattern of both gK and UL20p with UL20p and gK colocalized in intracellular compartments that stained for the TGN marker TGN46. Overall, these results showed that gK and UL20p intracellular transport and TGN localization were functionally interdependent strongly suggesting that gK and UL20p physically interacted [31]. Similar confocal colocalization assays were performed to test the ability of each UL20 mutant to facilitate transport and colocalization with gK. The CL38-CL49, Y38-Y49, Y38A and Y49A UL20 mutants produced similar patterns to those of the wild-type UL20 gene, since they effectively colocalized with gK (Fig. 4: rows 1–3). In addition, gK was colocalized with TGN46 (Fig. 4: rows 4–6), indicating that these UL20 mutations did not affect intracellular transport and TGN colocalization of the mutant UL20ps with gK. Similar assays were performed for the UL20p carboxyl terminal truncations 216t, 211t, and 204t (Fig. 5). The UL20p mutants CL153 and CL61 that were previously shown not to complement for either infectious virus production or virus-induced cell fusion [30] were also tested as negative controls, while the wild-type UL20 gene served as the positive control. Both 216t and 211t UL20 truncations enabled efficient colocalization of UL20 and gK in TGN compartments, while the 204t UL20 truncation failed to transport and colocalize with gK in TGN compartments (Fig. 5). Figure 5 represents a three color confocal microscopy experiment, while Figure 4 was a two-color confocal microscopy experiment.

### The effect of UL20 carboxyl terminal truncations on UL20p and gK TGN localization after endocytosis from cell surfaces

**Figure 6:**
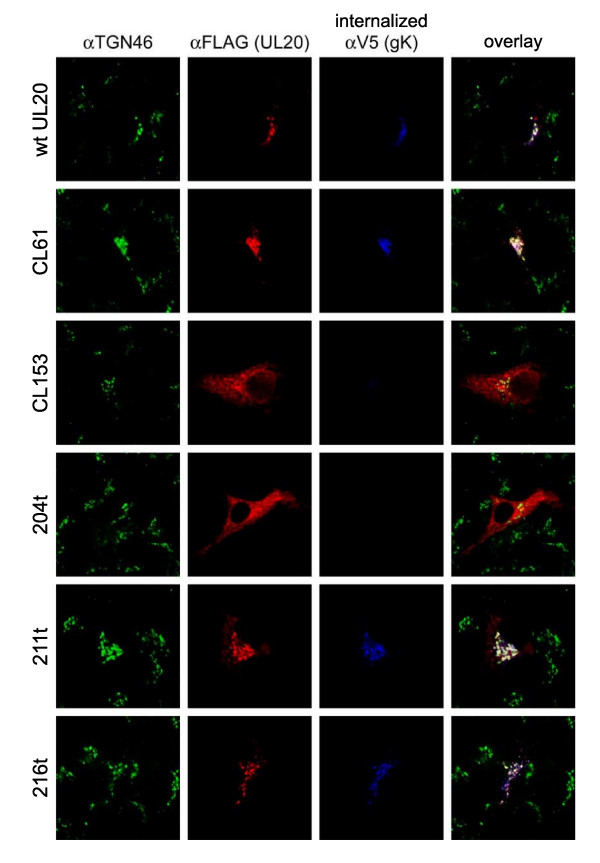
**Confocal microscopy of gK cell-surface expression and endocytosis to the TGN mediated by selected UL20p mutants**. Vero cells were co-transfected with gKD1V5 as well as with plasmids encoding wild-type or mutant UL20p, as with figures 4 and 5. Twenty-four hours post-transfection, cells were incubated under live conditions with aV5 (gK) mAb for 6 hours. Cells were washed thoroughly, fixed, and processed for confocal microscopy. After permeabilization, antibodies a3 × FLAG, aV5 and aTGN46 were used to identify, UL20p, gK and TGN46, respectively.

We reported previously that UL20 and gK are co-expressed on infected cell surfaces and co-internalize to TGN cytoplasmic membranes. Similar findings were produced in transient co-transfection experiments with both UL20 and gK genes [31]. Similar endocytosis assays were performed for the UL20p carboxyl terminal truncations. Briefly, in these experiments, Vero cells that coexpressed gK and UL20p were reacted with anti-V5 antibody under live conditions (see Materials and Methods). The fate of the internalized V5-tagged gK and FLAG-tagged UL20ps was assessed at different times post-labeling. By 6 h post-labeling, wild-type gK and UL20p labeled under live conditions on the transfected cell-surfaces were internalized and colocalized with TGN compartments (Fig. 6). The 216t, 211t, and CL61 mutants produced similar colocalization profiles of UL20p with gK in TGN membranes, while 204t and CL153 failed to colocalize UL20p and gK to TGN membranes following endocytosis (Fig. 6).

## Discussion

We showed previously that UL20 and gK are functionally interdependent for their intracellular transport, cell-surface expression and TGN localization [31] and that this interaction plays pivotal role in cytoplasmic virion envelopment and egress from infected cells [27]. In this study, we investigated the ability of selected UL20 mutations reported previously, as well as a new set of UL20 mutants, on their ability to transport and colocalize with gK on cell-surfaces and in TGN-labeled intracellular compartments:

Previously, we characterized a series of carboxyl terminal truncations including the 204t and 211t encoding carboxyl terminal truncations of 18 and 11 aa respectively. These two UL20p truncations failed to complement for infectious virus production and virus-induced cell fusion, while the 216t coding for a 6 aa truncation enabled virus-induced cell fusion, but failed to complement for infectious virus production [30]. We show here that the inability to complement for virus-induced cell fusion was not due to defects in intracellular transport and TGN localization, because 216t, as well as both 204t and 211t were efficiently transported to cell-surfaces and co-localized with gK in TGN-labeled membranes. Therefore, intracellular transport, cell-surface expression and TGN localization of UL20p and gK are not sufficient for infectious virus production. Based on these results, we can conclude that the carboxyl terminal six amino acids of UL20p function exclusively in intracellular virion envelopment and infectious virus production, while the UL20p domain spanning amino acids 204–211is important for both intracellular transport and virus-induced cell fusion.

Domain I is the largest domain (63 aa) and it includes stretches of acidic amino acid (D, E) clusters, which could form electrostatic interactions with other proteins [30]. Furthermore, the amino terminus of UL20p contains acidic clusters, as well as the amino acid motif YXXΦ (YSRL), which have been shown to function in endocytosis of alphaherpesvirus envelope proteins from plasma membranes to the TGN [32-36]. The acidic cluster motifs appear to direct TGN localization by binding to a cellular connector protein, PACS-1, which connects the glycoproteins to the AP-1 complex [37], while the YXXΦ motif binds adaptor proteins directly [2,3,40]. The YXXΦ (YSRL) amino acid sequence overlapping the CL49 mutated sequence, is conserved in HSV-1, HSV-2, and cercopithecine herpesvirus 1 and 2, but not in varicella zoster (VZV) or pseurodabies virus (PRV) (not shown). Mutagenesis of the Y residue of a YXXΦ(YTKI) motif within gK domain IV, shown to lie in the cytoplasmic side of membranes, produced a gK-null phenotype [20]. Similarly, mutagenesis of either Y38 or Y49, or both residues resulted in loss of infectious virus production, while the UL20p mutants carrying either mutation or a combination of both mutations allowed efficient intracellular transport and TGN localization. This result is similar to the results obtained with the UL20p carboxyl terminal domains and suggests that amino terminal domains of UL20p that function in cytoplasmic virion envelopment can be functionally separated from those that function in UL20p and gK intracellular transport and TGN localization. Interestingly, the Y49A mutant allowed some virus-induced cell fusion caused by either the gBsyn3 or gKsyn1 mutation suggesting that the requirement of this residue for infectious virus production is more stringent that the requirement for virus-induced cell fusion.

We reported previously that the Y49A, CL49 and 216t mutant viruses produced syncytial plaques, although their ultrastructural phenotypes seemed to be similar to that of the UL20-null virus [30]. We show here these phenotypes are consistent with the findings that these UL20p mutations allowed efficient intracellular transport, cell-surface expression and TGN localization. However, mutagenesis of both Y38 and Y49 amino acid residues in the amino terminus of UL20p, inhibited virus-induced cell fusion, while allowing efficient intracellular transport and TGN localization. This result suggests that the Y38 and Y49 residues together play important roles in cytoplasmic virus envelopment, but they are not required for proper UL20p/gK intracellular transport. The Y38A mutation seemed to affect both virion production and virus-induced cell fusion, although the Y49A mutation appeared to inhibit virion production, but allowed some cell fusion to occur. As is the case with the carboxyl terminus of UL20p discussed earlier, these results suggest that the amino terminus of UL20p contains functionally separable domains involved in cytoplasmic virion envelopment and intracellular glycoprotein transport. Furthermore, the Y49A mutation allowed some virus-induced cell fusion, but not infectious virus production to occur suggesting that domains within the UL20p amino-terminus involved in cytoplasmic virion envelopment may be functionally separated from domains functioning in UL20p/gK intracellular transport and virus-induced cell fusion.

## Conclusion

These results show that UL20p domains required for UL20p and gK intracellular transport and TGN localization can be functionally segregated from domains involved in infectious virus production and virus-induced cell fusion. The results suggest that virus-induced cell fusion mechanisms are not required for cytoplasmic virion envelopment.

## Materials and methods

### Cells and viruses

African green monkey kidney (Vero) cells were obtained from ATCC (Rockville, MD). The Vero-based UL20 complementing cell line, G5, was a gift of Dr. P. Desai, (John Hopkins Medical Center) [38]. Cells were maintained as previously described [20,29,38]. The parental wild-type strain used in this study HSV-1 (KOS) was originally obtained from P. A. Schaffer (Harvard Medical School). Δ20DIV5, Δ20gBsyn3 and Δ20gKsyn1DIV5 viruses were as described previously [27]. Virus stocks were grown on the UL20 complementing cell line Fd20-1, the construction of which was described previously [30]. In this paper, for simplification purposes, the Δ20DIV5 virus is referred to as Δ20 virus and the Δ20syngK1DIV5 virus is referred to as Δ20gKsyn1 virus [30].

### Plasmids

pCR2.1-UL20, which was used as the parental vector for UL20 mutagenesis, was generated by cloning a 773 bp DNA fragment containing the UL20 gene, obtained by PCR amplification of HSV-1(KOS) viral DNA, into pCR2.1/TOPO (Invitrogen) as described in detail previously [30]. The generation of UL20 cluster to alanine mutants CL38, CL49, CL153, and CL209, the single point mutant Y49A, and truncation mutants, 204t, 211t, 216t were reported previously [30]. A set of new UL20 mutants generated for this study included a UL20 mutant containing both the CL38 and CL49 mutations (CL38 – CL49), the alanine cluster UL20 mutant CL61, the single point mutant Y38A, and the UL20 mutant Y-Y containing both the Y38A and Y49A mutations. The cluster mutations, the additional single point UL20 mutants, as well as the double mutants were generated by splice-overlap extension (SOE) PCR [39] as described previously [30]. All mutations were verified by sequencing of the final plasmid construct.

### UL20 complementation assay for infectious virion production

Confluent Vero monolayers in six well plates were transfected with 2 μg of wild-type or mutant UL20 plasmid with Lipofectamine 2000 as described by the manufacturer (Invitrogen). Six hours post-transfection, the monolayers were infected with a UL20-null virus at an MOI of 1. Infections were placed on a rocker for 1 hour at 4°C, and then transferred to 37°C for 2 hours. Residual virus was inactivated using an acid wash (PBS containing .5 M glycine, pH3) for 2 min, and monolayers were subsequently washed 3 times with DMEM to restore the pH to a normal level. Infections were incubated at 37°C for 24 hours. After repeated freeze/thaw cycles, virus stocks were titered in triplicate on Fd20-1 cells, which effectively complement the UL20-null defect [30]. The complementation ratio for each mutant was calculated with the formula (virus titer of mutant/virus titer of negative control).

### UL20 complementation assay for virus-induced cell-to-cell fusion and virus spread

The complementation assay was performed essentially as we described previously for addressing the role of the HSV-1 UL11 protein in virion morphogenesis [40]. Briefly, confluent Vero monolayers in six-well plates were transfected with 2 μg of wild-type or mutant UL20 plasmid with Lipofectamine 2000 as described by the manufacturer (Invitrogen). 18 hours post transfection, the monolayers were infected at an MOI of 0.1 with either Δ20gKsyn1 or Δ20gBsyn3 viruses. Infections were placed on a rocker at room temperature for 1 hour, then transferred to 37°C for 30 minutes. Cells were overlaid with DMEM containing 1% methylcellulose. 24 hours post-infection, cell fusion was determined by visualization of syncytia formation by light microscopy. Cells were stained with a polyclonal HRP conjugated HSV-1 antibody as directed by the manufacturer (DakoCytomation). Briefly, cells were washed with PBS to remove methylcellulose media, and fixed with 4°C methanol for 15 minutes. TBS containing a 1:750 dilution of the polyclonal HSV-1 antibody was added to the cells and placed on a rocker at 4°C for 1 h. Cells were washed with TBS and developed using the Vector NovaRED peroxidase substrate kit as directed by the manufacturer (Vector, Inc). In this assay,

Complementation of the UL20-null virus by transient expression of the wild-type UL20 gene caused the production of up to 40% of total viral plaques appearing to have similar morphology and size to the HSV-1(F) parental virus.

### Confocal microscopy

Cell monolayers were grown on coverslips in six-well plates. Cell monolayers were transfected with the indicated UL20 and/or gK plasmid combinations by using Lipofectamine 2000 (Invitrogen) according to the manufacturer's instructions and prepared for confocal microscopy approximately 30 h posttransfection. Cells were washed with TBS and fixed with electron microscopy-grade 3% paraformaldehyde (Electron Microscopy Sciences, Fort Washington, Pa.) for 15 min, washed twice with phosphate-buffered saline-50 mMglycine, and permeabilized with 1.0% Triton X-100. Monolayers were subsequently blocked for 1 h with 7% normal goat serum and 7% bovine serum albumin in TBS (TBS blocking buffer) before incubation for 2 h with either anti-V5 (Invitrogen, Inc.), for recognition of gK, or anti-FLAG (Sigma Chemical, Inc.), for recognition of UL20p, diluted 1:500 in TBS blocking buffer. Alternatively, simultaneous detection of gK and UL20p in cotransfected cells was accomplished by concurrent incubation with murine anti-V5 and rabbit anti-FLAG (Sigma Chemical, Inc.) diluted 1:500 in TBS blocking buffer. Cells were then washed extensively and incubated for 30 min with Alexa Fluor 594 and/or Alexa Fluor 647-conjugated anti-immunoglobulin G diluted 1:500 in TBS blocking buffer. After incubation, excess antibody was removed by washing five times with TBS. TGN were identified with a donkey anti-TGN46 primary antibody and an Alexa Fluor 488-conjugated sheep anti-donkey secondary antibody [41]. Specific immunofluorescence was examined using a Leica TCS SP2 laser scanning confocal microscope (Leica Microsystems, Exton, Pa.) fitted with a CS APO 63× Leica objective (1.4 numerical aperture). Individual optical sections in the *z *axis, averaged six times, were collected at the indicated zoom in series in the different channels at 1,024- by 1,024-pixel resolution as described previously [27,29,42]. Images were compiled and rendered with Adobe Photoshop. Image analyses were generated and analyzed using the Leica confocal microscopy software package and were modified from protocols described previously [43].

### UL20p/gK cell surface internalization assay

Internalization assays were modified from similar assays performed previously [35,44,45]. Briefly, Vero cells were transfected with pgKDIV5 and either pUL20-3 × FLAG or a variant containing the indicated UL20 mutation [29]. Twenty hours posttransfection, cells were incubated under live conditions for 6 h at 37°C with mouse anti-V5. Cells were extensively washed, fixed with paraformaldehyde, and processed for confocal microscopy as described above, with the exception that the internalized antibodies served as the primary antibody for gK (mouse anti-V5).

## Competing interests

The author(s) declare that they have no competing interests.

## Authors' contributions

J. Melancon performed most of the experiments. K. G. Kousoulas wrote the manuscript.
